# Wearable Sensor Based on Flexible Sinusoidal Antenna for Strain Sensing Applications

**DOI:** 10.3390/s22114069

**Published:** 2022-05-27

**Authors:** Mehran Ahadi, Mourad Roudjane, Marc-André Dugas, Amine Miled, Younès Messaddeq

**Affiliations:** 1 Center for Optics, Photonics and Lasers (COPL), Department of Electrical and Computer Engineering, Université Laval, Quebec City, QC G1V 0A6, Canada; mehran.ahadi.1@ulaval.ca; 2 LABioTRON Bioengineering Research Laboratory, Department of Electrical and Computer Engineering, and Research Centre for Advanced Materials (CERMA), Université Laval, Quebec City, QC G1V 0A6, Canada; amine.miled@gel.ulaval.ca; 3 Center for Optics, Photonics and Lasers (COPL), Department of Physics, Université Laval, Quebec City, QC G1V 0A6, Canada; mourad.roudjane.1@ulaval.ca; 4 Département de Pédiatrie, Faculté de Médecine, Centre Mére-Enfant Soleil du CHU de Québec, Université Laval, and Centre de Recherche du CHU de Québec, Quebec City, QC G1V 4G2, Canada; marc-andre.dugas.med@ssss.gouv.qc.ca

**Keywords:** dipole antenna, miniaturized antenna, sinusoidal antenna, strain sensor, tunable antenna, conductive polymer, antenna sensor

## Abstract

A flexible sinusoidal-shaped antenna sensor is introduced in this work, which is a modified half-wave dipole that can be used for strain sensing applications. The presented antenna is an improved extension of the previously introduced antenna sensor for respiration monitoring. The electrical and radiative characteristics of the sinusoidal antenna and the effects of the geometrical factors are studied. An approach is provided for designing the antenna, and equations are introduced to estimate the geometrical parameters based on desired electrical specifications. It is shown that the antenna sensor can be designed to have up to 5.5 times more sensitivity compared to the last generation of the antenna sensor previously introduced for respiration monitoring. The conductive polymer material used to fabricate the new antenna makes it more flexible and durable compared to the previous generation of antenna sensors made of glass-based material. Finally, a reference antenna made of copper and an antenna sensor made of the conductive polymer are fabricated, and their electrical characteristics are analyzed in free space and over the body.

## 1. Introduction

The electrical and radiative characteristics of antennas are functions of their geometrical structure and the material specifications of the conductors and dielectrics in the antenna’s vicinity [1]. Consequently, antennas can be exploited for sensing applications, where the geometrical deformations or material changes can be detected by monitoring the antenna’s radiative and electrical characteristics. Applications of antenna sensors can be in body movement capturing for computer animation or robot controlling, monitoring structural deformations, such as tracking cracks and displacements in buildings, monuments, and similar structures, and most importantly, monitoring vital signals for diagnosis and rehabilitation [2,3,4,5,6]. A Serpentine meshed patch antenna is reported for stretch strain sensing fabricated using laser-cut conductive textiles on Ecoflex substrate [7]. Recently, an RFID-incorporated meandered line dipole antenna in Ecoflex has also been reported to detect stretching strain [8].

In addition, some recently published works are focused on detecting bending strain. For example, Graphene-based patch antennas are introduced in recent works for detecting bending strain with applications in identifying human posture and joint movements [9,10]. An aluminum tape patch over a cellulose substrate is also reported in another work for bending strain detection. The use of cellulose makes the antenna recyclable and suitable to employ in disposable electromechanical sensors [11]. Some other works are focused on structural health monitoring (SHM) applications. Recent works in this area are in the form of rectangular [12], circular [13], and folded patch antennas [14]. Fractal-shaped patch antennas are also studied by Herbko and Lopato for a more miniaturized SHM antenna sensor [15]. More recently, a novel metamaterial-based SHM antenna sensor was also reported by the same team using a double split-ring resonator (dSRR) structure to achieve even more miniaturization while increasing the strain sensitivity [16].

Antenna sensors can also be embedded in wearables for vital signal monitoring applications. Although many sensing technologies have been developed for monitoring vital signals [17,18], applying on-body antenna sensors for this purpose is a relatively new field of study. A spiral-shaped flexible dipole antenna was reported for respiration detection by analyzing the received signal strength indicator (RSSI) of a Bluetooth connection [2]. Recently, a low-profile fully textile patch antenna was proposed for respiration monitoring applications [19]. A new antenna sensor in the form of a fully embroidered meander line dipole was also proposed in a recent work for real-time respiration monitoring [20].

This work presents a flexible sinusoidal-shaped half-wave dipole antenna sensor that can be used in strain sensing and vital signal monitoring applications. The proposed sinusoidal antenna sensor is the new generation of a previously introduced antenna sensor for respiration monitoring [2]. Using flexible conductors as antenna material, the changes in the antenna’s impedance due to the mechanical compressions or stretchings can be picked up by a measurement device as an indicator of the strain applied to the antenna [4]. It is shown that the antenna sensor introduced in this paper could be up to 5.5 times more sensitive than the previous generation [2].

The sinusoidal antenna introduced in this paper is a modified version of the half-wave dipole antenna. Modified dipoles are reported and studied in the literature for the sake of miniaturization and impedance control, such as meandered, zigzag [21,22,23,24], and monopole sinusoidal geometries [25,26]. They are also employed in Log Periodic Dipole Array (LPDA) antennas [27,28] and Radio frequency identification (RFID) tags [29,30] due to their short axial length. Similarly, the sinusoidal antenna introduced here has a resonant impedance lower than the traditional straight dipole antenna and an axial length shorter than λ/2. For example, a sinusoidal antenna designed for 50 Ω is 20% axially shorter than a traditional straight half-wave dipole. The miniature size of the sinusoidal antenna makes it suitable for wearable applications.

Our implemented antenna sensor for vital signal monitoring is designed to be placed over the front of the chest area and embedded in a T-shirt [4]. This method of vital signal monitoring is different from non-contact systems, where both antenna and the measurement and detection system are located remotely from the subject [17]. The changes in the circumference of the upper body may lead to a geometrical deformation of the wearable antenna sensor, which changes its radiative and electrical characteristics, which will be detected by a measurement system. Sensitivity to strain, stretchability, flexibility, and durability are crucial factors for achieving a viable vital signal sensing system, as well as the Specific Absorption Rate (SAR) since these antenna sensors are placed over the body.

In the light of these requirements, this paper paves the way for realizing a vital signal monitoring system with a more sensitive, flexible, durable, and miniaturized antenna sensor compared to the previous generation [2,3,4]. A detailed study of the sinusoidal antenna’s radiative and electrical specifications for various configurations is provided, its sensitivity to deformations is studied, and a design guide is provided for the antenna sensor. Finally, a prototype antenna is fabricated and measured in free space and over the body, and its durability and SAR compliance are studied. An upcoming paper will cover the antenna’s application in a vital signal monitoring system and provide measurement data for the antenna sensor.

The sinusoidal antenna is introduced and studied in detail in the second and third sections of this paper. The antenna is simulated using CST Studio Suite^®^ 2020 software [31], and the effects of the geometrical parameters are analyzed on its radiation characteristics and strain sensitivity. A design guide is provided in the fourth section using two methods, and equations are introduced for estimating the geometrical parameters based on desired characteristics. In the fifth section, a reference copper antenna and a flexible polymer antenna sensor are fabricated, their electrical parameters are measured, and their environmental durability is analyzed through an experiment. Discussions are made in the sixth section about the antenna’s SAR, its behavior under bending and twisting, and its application in strain sensing. Additionally, a summary of the state-of-the-art is provided, and it is shown that higher sensitivity to strain is achievable using this antenna sensor compared to the traditional design used in our previous work, known as a half-turn Archimedean spiral antenna [2].

## 2. Antenna Geometry

**Figure 1:**
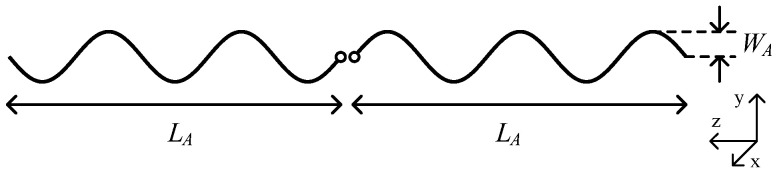
Antenna Geometry with n=5. Small circles in the middle indicate the feeding point. The antenna is placed along *Z*-axis, and the peaks and dips are along the *Y*-axis.

The design of the antenna sensor, shown in Figure 1, is based on the traditional straight half-wave dipole. The antenna wires are bent n times to form a sinusoidal shape in the domain of zero to nπ. Equation (1) can be considered for plotting the shape of a single pole of the antenna in a cartesian coordinate system where parameters $W_{A}$ and $L_{A}$ define the width and the length of the antenna, respectively. The n factor indicates the number of extrema in the structure of a single antenna pole.

(1) $$\begin{array}{c} y=W_{A}\sin\left(\frac{n\pi }{L_{A}}z\right)\;z=\left(0,L_{A}\right) \end{array}$$

## 3. Specifications and Intrinsic Parameters

A straight half-wave dipole antenna has a total wire length of 2LW=λ2 relative to its wavelength of operation, and ideally has an inductive impedance of $Z_{W}=73.1+43j$ on the respective frequency [32]. For the modified half-wave dipole antennas, the Shortening Ratio (SR) is defined as the ratio between the reduction in its axial length and its total wire length, which is shown as [21,25,26]:

(2) SR=λ2−2LAλ2=1−LA LW 

The antenna introduced here is simulated using CST Studio Suite^®^ 2020 full-wave electromagnetic simulation software [31]. The antenna is modeled by very thin wires made of Perfect Electric Conductor (PEC) and with a diameter d of less than $\lambda \times 10^{-4}$ as an approximation of an infinitesimally thin antenna [1]. A feeding point gap of $L_{g}=1.25\times 10^{-5}\lambda$ is considered to keep it as small as possible. The model details mentioned here are considered for all of the simulations presented in this work.

**Figure 2:**
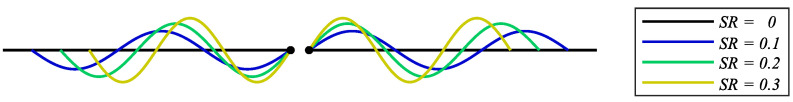
Sample design of an antenna with n=3 for different values of SR. It is evident that the more the SR increases, the higher the width of the antenna.

**Figure 3:**
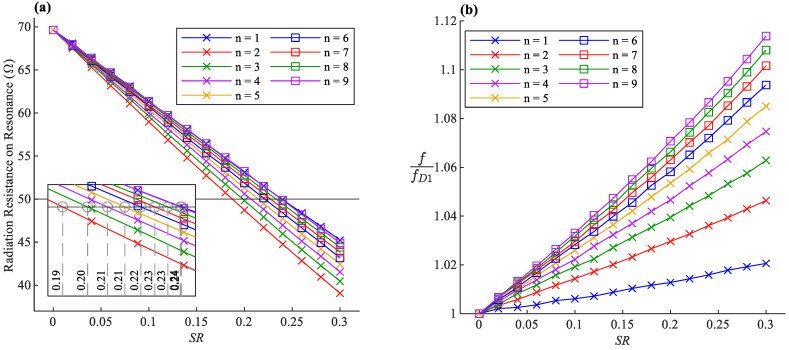
(**a**) Radiation Resistance of the antenna on resonance frequency. Inset: zoomed-in detail of the crossing around the 50 Ω line. (**b**) The resonance frequency of the antenna normalized to the resonance frequency of the straight half-wave dipole ($f_{D1}$ ).

A set of calculations are made on the introduced antenna geometry for n=1,…,9, in which the SR is increased in fine steps while keeping wire length ($L_{W}$) constant, as shown in Figure 2. The antenna’s width has to increase in each step to ensure the constancy of the wire length. The simulation results are presented in Figure 3. The antenna’s resonance frequency is normalized to the resonance frequency of the straight half-wave dipole ($f_{D1}$). It is evident that the more the antenna becomes compressed, the lower the radiation resistance on resonance, but the higher the resonance frequency.

### 3.1. Radiation Resistance

The decrease of radiation resistance due to the increase of SR seen in Figure 3a is expected behavior. To analyze the radiation resistance, we need to observe the antenna from a far-field point of view, with a distance of r from the antenna, where r≫λ. With the assumption of $W_{A}\ll \lambda$, the oscillations of the sinusoidal shape can be ignored from a far-field standpoint. Therefore, the antenna can be approximated as a straight dipole with a physical length of $2L_{A}$ which is less than half of the operation wavelength. The antenna seen from the far-field is electrically shorter than a half-wave dipole. The real part of its impedance, indicating the radiation resistance, will be less than $ℜe\;\left\{Z_{w}\right\}$ which is expected to become even smaller as it becomes a shorter dipole [1,21].

### 3.2. Resonance Frequency

The normalized resonance frequency of the antenna is shown in Figure 3b as a function of SR for the different number of bents (n parameter). The point SR=0 is equivalent to a straight half-wave dipole $L_{A}=L_{W}$, and therefore the simulation results show a resonance at around $R_{D1}\approx 70\;\Omega$ and a normalized frequency of unity. While the wire length is kept constant, the resonance frequency increases as the SR rises, which means that the rise of peaks and dips in antenna geometry causes an extra capacitive effect on the antenna [21].

### 3.3. Effects of the n Factor

It can be concluded from Figure 3a that the higher the n factor, the shorter the axial length of the antenna for specific radiation resistance, making it more miniaturized. Additionally, the antenna’s sensitivity to the deformations becomes more significant by choosing higher n values.

**Figure 4:**
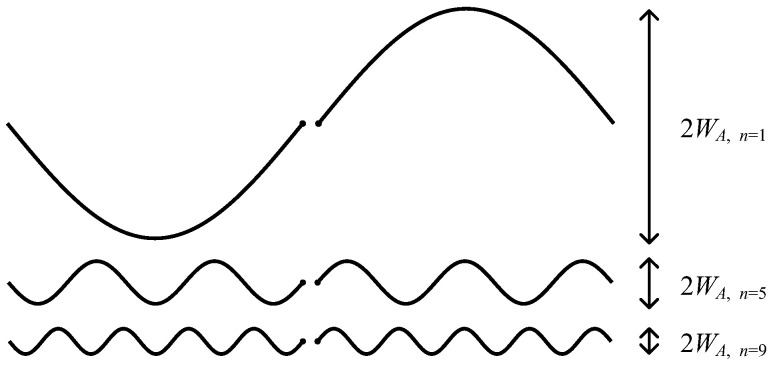
Antennas designed for radiation resistance of 50 Ω on a specific frequency using values of 1, 5, and 9 for the n factor. The higher the n factor, the less the antenna width for the same resonance frequency and radiation resistance, resulting in a more linear structure.

In the case of n=1, the antenna becomes so wide that it violates the assumption of $W_{A}\ll \lambda$ and cannot be categorized as a linear dipole antenna from a far-field point of view. For example, the required designs to achieve 50 Ω using n=1, 5, and 9 are shown in Figure 4. The width of the antenna n=1 is noticeably large and around ≈0.15λ, which is violating $W_{A}\ll \lambda$. Consequently, by choosing n=1, the antenna cannot be approximated with a linear equivalent antenna from the far-field point of view anymore. The antenna would virtually become a superposition of two perpendicular equivalent dipoles with specifications out of this work’s context.

### 3.4. Radiation Pattern and Maximum Gain

**Figure 5:**
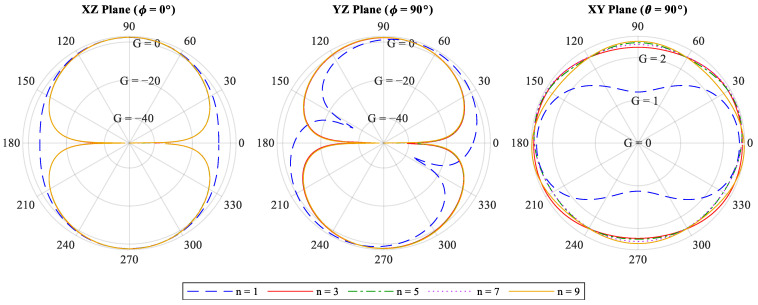
Far-field radiation gain (G) pattern of the 50 Ω  sinusoidal dipole antenna for *n*=1,3,…,9. The antenna is placed along *Z*-axis, and the sinusoidal peaks and dips are spread along *Y*-axis.

The radiation patterns of antennas designed for 50 Ω are illustrated in Figure 5. The antennas designed with the constraints of n>1 have a radiation pattern similar to a straight half-wave dipole. The maximum gain and Half-power beamwidth (HPBW) are presented in Table 1 for a straight half-wave dipole antenna (n=0) and sinusoidal antennas $R_{R}=50\;\Omega$ and n=1, 3,…, 9. It can be concluded that a larger n makes the antenna more similar to the straight half-wave dipole antenna in terms of HPBW and far-field pattern. The HPBW is slightly wider than the straight dipole in lower n values and becomes narrower as n rises, resulting in more directivity (D) and maximum gain (G), as G∝D [1]. Following the discussion above about the effects of the n factor, it can be seen that the unusual radiation pattern for the case of an antenna n=1 resembles a superposition of two dipoles along the *Z*- and *Y*-axis due to its noticeably large width of $W_{A}\approx 0.15\lambda$.

## 4. Design Methods

Here a set of curve fittings are made of the simulation data to provide equations based on geometrical parameters for estimating the impedance on resonance and the resonance frequency of the sinusoidal antenna, which can provide a starting point for fine-tuning the antenna parameters. Two methods are presented, based on two constraints of fixed wire length or fixed axial length, and the pros and cons of each are described. The curve fittings are made through a Nonlinear Least Squares (NLS) method based on the Trust Region algorithm [33].

### 4.1. Method 1: Designing Based on $L_{A}$ for a Known $L_{W}$

#### 4.1.1. Curve Fittings Based on SR

In the data presented in Figure 3, the radiation characteristics are expressed based on SR, which is the antenna length $L_{A}$ relative to the fixed total wire length $L_{W}$. According to the curve fittings, the resonance frequency f increases with the rise of SR, and the slope is proportional to the square root of n. It can be written in the following form:

(3) $$\begin{array}{c} f=f_{D1}\;\left(1+\frac{\sqrt{n}}{k_{11}}\cdot SR\right) \end{array}$$

where $f_{D1}$ is the resonance frequency of a straight half-wave dipole with a length of $L_{W}$. The impedance on resonance $R_{R}$ drops as SR increases, and the drop rate is approximately proportional to the 8th root of n. Therefore, it can be fitted on the following form:

(4) $$\begin{array}{c} R_{R}=R_{D1}\left(1-\frac{k_{12}}{\sqrt[{8}]{n}}\cdot SR\right) \end{array}$$

**Figure A1:**
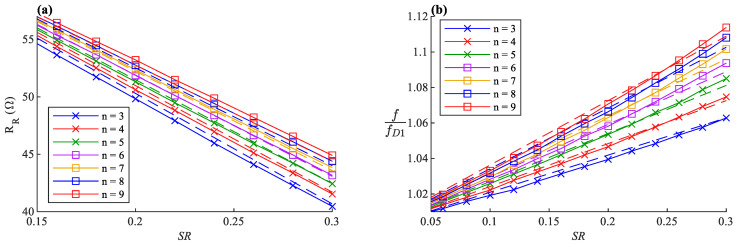
Correlation of the data and the equations achieved via curve fitting based on SR for (**a**) radiation resistance on resonance frequency (zoomed in) and (**b**) resonance frequency normalized to $f_{D1}$. Solid lines represent the data, and dashed lines represent the fitted function. It is clear that the fitted function on $R_{R}$ is ambiguous for adjacent n values.

By choosing $k_{11}=8.265$ in Equation (3), and $R_{D1}=69.54,\;k_{12}=1.587$ in Equation (4), an $R^{2}$ of 99.55% and 99.85% is achieved for each Equation, respectively, as a measure of goodness-of-fit [34]. The curve fitting is made of the data in the domain of n=3,…,9 and SR=0.05,…, 0.3. The precision of the curve fittings is shown in Figure A1 in Appendix A. It is also possible to choose a $R_{R}$ or f of choice and solve the introduced equations for SR. It should be noted that n factor can only take integer values and should be chosen according to the fabrication capabilities.

#### 4.1.2. The Design Steps Using Method 1

1. Choosing a wire length $L_{W}$ and n
2. Calculating $R_{R}$ for different SR values, or calculate SR for a given $R_{R}$
3. Calculating f for the SR chosen in step 2
4. Readjusting $L_{W}$ (and subsequently updating $f_{D1}$) while keeping SR fixed to reach the desired resonance frequency
5. Calculating $W_{A}$ based on the finalized $L_{A}$ and $L_{W}$ using the integral Equation introduced in the following.
6. Verify the design by simulating the antenna model based on Equation (1)
7. Finish if the desired frequency is acquired; otherwise, repeat from Step 4.

It is worth mentioning that the designer has to redo all the steps if they decide to choose another n value.

#### 4.1.3. Calculating Antenna width $W_{A}$

With $L_{W}$ and $L_{A}$ as known variables, calculating the width of the antenna ($W_{A}$) requires solving an integral equation. A small arc dL can be fit on the hypotenuse of a right triangle. According to the Pythagorean theorem, its length can be written as $dL=\sqrt{dy^{2}+dz^{2}}$. By integrating dL over the curve, the total curve length can be calculated as follows:

(5) $$\begin{array}{c} L=\int_{a}^{b}\sqrt{1+{\left(\frac{dy}{dz}\right)}^{2}}dz. \end{array}$$

By combining (1) and (5), the wire length can be calculated as the following:

(6) $$\begin{array}{c} L_{W}=\int_{0}^{L_{A}}\sqrt{1+{\left(W_{A}\frac{n\pi }{L_{A}}\;\cos\left(\frac{n\pi }{L_{A}}z\right)\right)}^{2}}dz\;. \end{array}$$

#### 4.1.4. Disadvantages of Method 1 Based on SR

The integral in Equation (6) does not have an elementary antiderivative and is an elliptic integral of the second kind. Even though numerical methods and software packages such as MATLAB can be used to solve the integral Equation for $W_{A}$ effortlessly [35,36], it would not be a convenient method in the antenna design synthesis process, especially if the design needs to be made based on antenna width $W_{A}$ parameter.

Although the definition of SR could help to understand the behavior of the antenna, it is not a decent choice for providing a design method since its dependency on constant wire length $L_{W}$ requires solving Equation (6) for $W_{A}$ before each iteration of the simulation to obtain the full geometrical parameters of the antenna. Additionally, the designer has to redo all the design steps in the case they decide to go with another value of n. Moreover, the estimation of $R_{R}$ using Equation (4) based on SR cannot differentiate well between adjacent n values since the data lines are very close.

### 4.2. Method 2: Designing Based on $W_{A}$ for a Known $L_{A}$

#### 4.2.1. Widening Ratio

Widening Ratio (WR) is presented in Equation (7) for studying the antenna characteristics directly based on the geometric parameters of $W_{A}$ and $L_{A}$ and alleviate the need for solving the integral equations during the antenna design process.

(7) $$\begin{array}{c} WR=\frac{W_{A}}{L_{A}}\times 100. \end{array}$$

**Figure 6:**
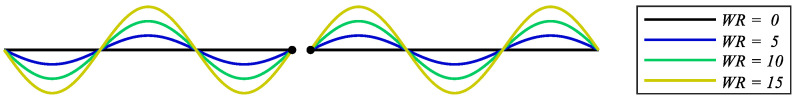
Representation of increasing WR of an example antenna with n=3, while keeping $L_{A}$ as constant and ignoring the fixed wire length $L_{W}$ constraint.

**Figure 7:**
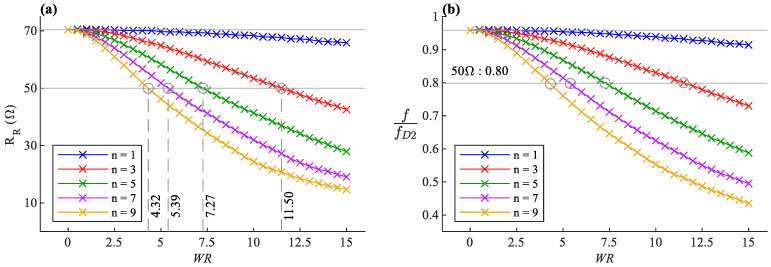
Antenna characteristics for different n values expressed versus WR while the axial length $L_{A}$ is kept constant (**a**) Radiation Resistance of the antenna on resonance frequency. The required WR values to design a 50 Ω antenna are marked for n=3,…,9 (**b**) Resonance frequency normalized to $f_{D2}$.

A new set of simulations were performed on antennas with different WR values while keeping $L_{A}$ constant and ignoring the assumption of fixed total wire length $L_{W}$. This definition is beneficial while modeling the antenna based on $L_{A}$ and $W_{A}$ in electronic design automation (EDA) software and electromagnetic simulators. Figure 6 shows an example representation of the simulated model configurations for n=3. This set of simulations are repeated for different n values, and the results are presented in Figure 7.

##### 4.2.2. Curve Fittings Based on WR

**Figure A2:**
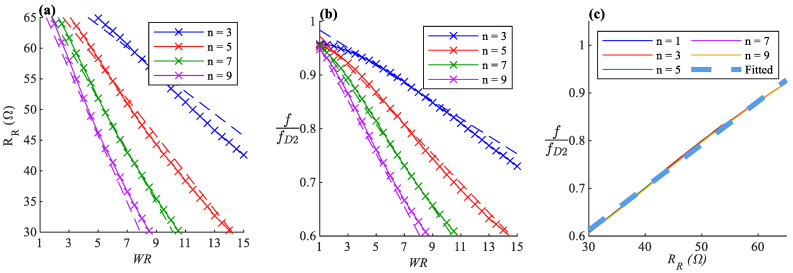
Correlation of the data and the equations achieved via curve fitting based on WR for (**a**) radiation resistance on resonance frequency and (**b**) resonance frequency normalized to $f_{D2}$. Solid lines represent the data, and dashed lines represent the fitted function. (**c**) Correlation of the data and Equation (10), it is evident that the data are almost independent of n factor. The dashed line shows the fitted Equation.

According to the data presented in Figure 7, the decrease in f and $R_{R}$ is approximately proportional to n and WR, and Equations (8) and (9) are provided based on this observation. These equations provide approximations for a given n and WR, as a starting point for fine-tuning. In this Equation $f_{D2}$ is the resonance frequency of a straight half-wave dipole with a length of $L_{A}$, and by setting $k_{21}=182.3$ in Equation (8) and $R_{D2}=72.78,\;\;k_{22}=120.94$ in Equation (9), the equations fit approximately on the data points with an $R^{2}$ of 98.41% and 99.07%, respectively. The precision of the fitting is illustrated in Figure A2a,b in Appendix A.

(8) $$\begin{array}{c} f=f_{D2}\;\left(1-\frac{n}{k_{21}}\cdot WR\right) \end{array}$$

(9) $$\begin{array}{c} R_{R}=R_{D2}\left(1-\frac{n}{k_{22}}\cdot WR\right) \end{array}$$

The surface fitting is made with the data in the domain of n=3,…,9, WR=1, …, 15 and the range of $R_{R}=30,\ldots ,\;65$ and $f/f_{D2}=0.6,\;\ldots ,\;1$. Similarly, these equations can also be solved for WR considering a f or $R_{R}$ of choice. By combining Equations (8) and (9), we reach the following Equation (10), which defines f solely based on $R_{R}$ and does not depend on n, as shown in Figure A2c in Appendix A.

(10) $$\begin{array}{c} f=f_{D2}\;\left(1-k_{31}\left(R_{D3}-R_{R}\right)\right) \end{array}$$

where:

(11) $$\begin{array}{c} k_{31}=\frac{k_{22}}{k_{21}},\;R_{D3}=\frac{k_{22}}{k_{21}}R_{D2}\; \end{array}$$

##### 4.2.3. The Design Steps Using Method 2

1. Choosing a wire length $L_{A}$ and n
2. Calculating $R_{R}$ for different WR values, or calculate WR for a given $R_{R}$
3. Calculating f for the WR chosen in step 2
4. Readjusting $L_{A}$ (and subsequently updating $f_{D2}$) while keeping WR fixed to reach the desired resonance frequency
5. Verify the design by simulating the antenna model based on Equation (1)
6. Finish if the desired frequency is acquired; otherwise, repeat from Step 4.

##### 4.2.4. Advantages of Method 2 Based on WR

This design method has three advantages compared to the previous method, based on SR. First, the design is made directly based on $L_{A}$ and $W_{A}$ values and no integrals need to be solved to calculate $W_{A}$ for each iteration of the design process. Second, the estimation of $R_{R}$ has a better separation for adjacent n values compared to the previous method. Third, if the designer finishes the design process and then decides to go with another n factor, they will not need to start over. By only repeating the second step, a new WR can be calculated for the previously chosen resistance, and then $W_{A}$ can be readjusted. As shown in Equation (10), the resonance frequency of the antenna will not change as long as the designer keeps the previously chosen radiation resistance for the design.

### 4.3. Tuning Considerations

The factors affecting classic straight dipole antenna characteristics also apply to the sinusoidal dipoles, such as feeding point gap width, wire conductivity, and wire thickness [32]. All simulations were achieved using PEC as the material of the antenna wires. For convenience, the designer might consider extending the feeding point gap of $L_{g}=1.25\times 10^{-5}\lambda$ to a larger value for high-frequency antennas where λ is shorter than a kilometer. In this case, the WR (or SR) factor needs to be adjusted to a slightly lower value for compensating the inductive effect caused by the extra gap widening.

All the data presented from the simulation runs in this work were prepared with the assumption of a very thin wire with a diameter d of less than $\lambda \times 10^{-4}$. For an antenna made of a thicker wire, the WR (or SR) parameter needs to be adjusted to a slightly higher value to achieve the desired radiation resistance on resonance.

## 5. Fabrication and Measurement

### 5.1. Fabrication Process

Two sample high-frequency antennas with n=5 are fabricated as a proof-of-concept. Table 2 shows the geometrical parameters used for the fabrication of both antennas. The antenna is designed and simulated for 800 MHz and 50 Ω impedance. A copper-made antenna is fabricated as the reference, and a second antenna is also made using a conductive flexible polymer suitable for wearable sensor applications.

Compared to the previously introduced traditional antenna sensor based on silica hollow-core fibers, the newly developed polymer fiber is enduring, flexible, and sustains its electrical characteristics better than silica hollow-core fibers [37]. The conductive polymer is biocompatible, highly flexible, and resistant to water and other perturbations. It can be easily sewn on textiles and bending, twisting, or stretching does not break it. The measured resistance of the material is around $\approx 8\;\Omega \cdot {\mathrm{cm}}^{-1}$ and is observed to have a good performance in high frequency.

The composition of the conductive polymer fiber is a combination of poly (ethylene-co-vinyl acetate) (PEVA) polymer (Sigma-Aldrich, St. Louis, MO, USA) and multi-walled carbon nanotubes (MWCNTs) (commercially available at Cheaptubes, Grafton, VT, USA, with a carbon purity of 95 wt%) with a composition of 41 wt% mass of MWCNT and 59 wt% mass of PEVA and without any purifications.

**Figure 8:**
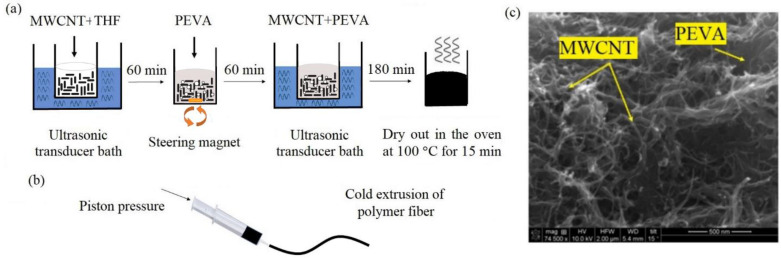
(**a**) Fabrication process of the MWCNT-PEVA conductive polymer (**b**) the extrusion process of the polymer wire (**c**) SEM image of the cross-section of the MWCNT-PEVA polymer wire.

The fabrication steps of the conductive polymer are shown in Figure 8a. For an hour, the MWCNT nanoparticles are sonicated in 10 mL of tetrahydrofuran (THF) (Fisher Scientific International, Waltham, MA, USA). Afterward, the PEVA polymer and the MWCNTs solution are mixed by mechanical stirring for one hour at 1400 rpm. The mixture is then sonicated for an additional 180 min for better dispersion. Finally, the colloidal solution is placed in the oven at 100 °C for 15 min to obtain a high-viscosity composite.

The composite of the conductive fiber is extruded [38] using a commercially available syringe, as shown in Figure 8b, and is left to dry out. The initial thickness of conductive fiber is 1.6 mm, which reduces to 1.4 mm after the drying process. The scanning electron microscopy (SEM) image of the cross-section of the polymer wire is presented in Figure 8c.

### 5.2. Measurements

**Figure 9:**
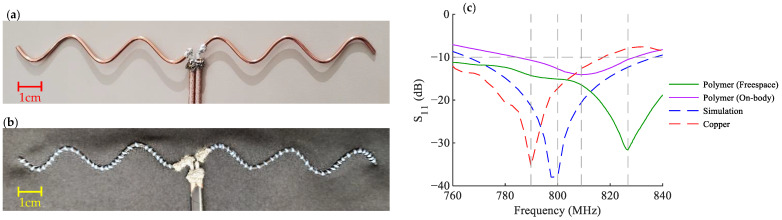
(**a**) The Copper antenna (**b**) The Polymer antenna, sewn on a T-shirt (**c**) Return loss of the antenna in simulation, the Copper antenna, and the Polymer antenna, both in free space and over the body.

The fabricated antennas are shown in Figure 9a,b and are fed using the commercially available 1:1 balun TC1-1-13MA+ from Mini-Circuits [39]. The measurements are made using a calibrated VNA system, with effects of the transmission line de-embedded from the final scattering parameters (S-Parameters) readout [40]. Table 3 shows the measured specifications of the fabricated antennas. The return loss of the antennas is also presented in Figure 9c. Each measurement is repeated eight times, and the average values are reported. The measurements show a good agreement between the antennas and the simulation. The differences are due to the error in fabrication, the material specifications, and non-rigid nature of the polymer wire.

It can be seen that the resonance frequency of the polymer antenna is changed when it is placed over the body. There are two factors contributing to this frequency shift. The first one is the changes in the antenna’s surrounding material in its reactive near-field region, which makes a change in the impedance of the antenna seen from the feeding circuit, resulting in an impedance mismatch and therefore affecting the resonance frequency and the radiation resistance of the antenna [41].

**Figure 10:**
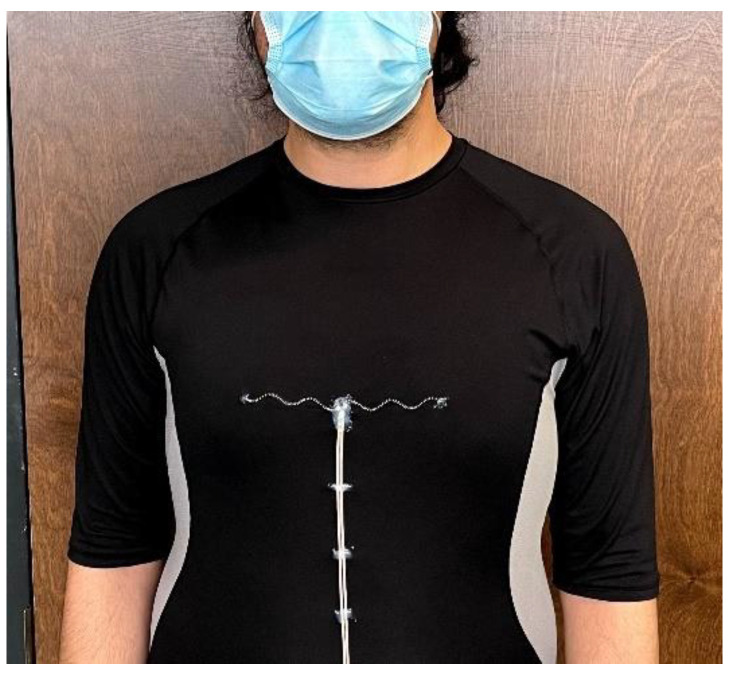
A 50 Ω antenna sensor n=5, embedded on a smart T-shirt for vital signal monitoring applications.

The other factor causing this shift is the deviation of the antenna’s dimensions due to its initial stretching when the patient wears the smart textile, as shown in Figure 10. Therefore, it is recommended that the T-shirt selected for the antenna integration be a right fit for the patient’s body form and not too tight. Otherwise, the initial state of the antenna sensor will be considerably stretched, which would potentially limit the dynamic range of the antenna sensor.

### 5.3. The Durability of the Antenna Sensor

An experiment is performed in order to demonstrate the durability of the conductive polymer material for wearable smart textile applications intended for everyday use. A 50 Ω antenna sensor is fabricated and sewn on a piece of fabric, and its electrical specifications are measured. The fabric and the antenna sensor are washed for 20 cycles, and the measurements are performed again after each cycle.

During each step, the fabric is submerged in a container filled with tap water and detergent and is stirred vigorously to simulate a washing procedure. The fabric is taken out after 5 min, and then it is rinsed using tap water. Next, a piece of a napkin is used to remove the excessive wetness of the fabric. Finally, a heat gun is used for 10 to 15 min to completely dry out the fabric and antenna sensor. The specifications of the antenna sensor are remeasured and recorded after the complete dry out.

**Figure 11:**
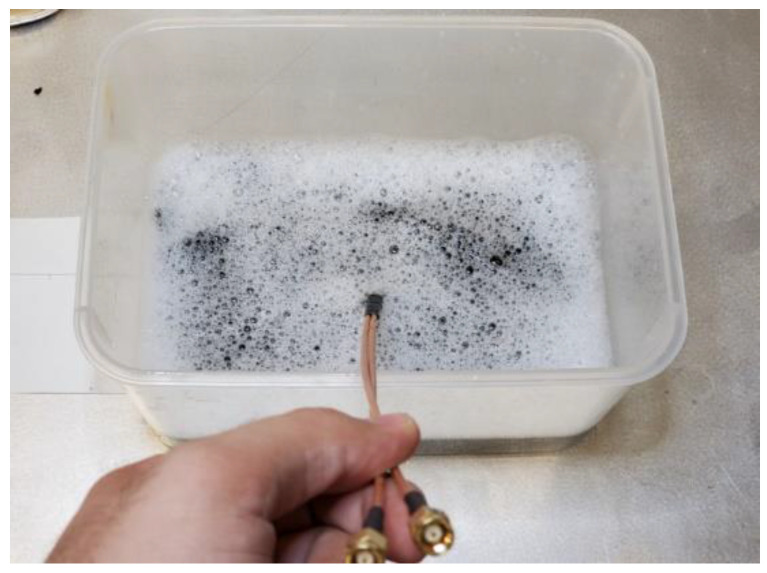
The fabric is completely submerged in water and detergent. The SMA connectors are held outside.

The container water and the detergent were refreshed every five cycles. The SMA connectors were held outside the water during the washing process, as shown in Figure 11. The heat gun is placed at least 15 cm away from the fabric during the drying process to prevent accidental burning due to its high temperature and is manually swung over the fabric to ensure complete dryness.

**Figure 12:**
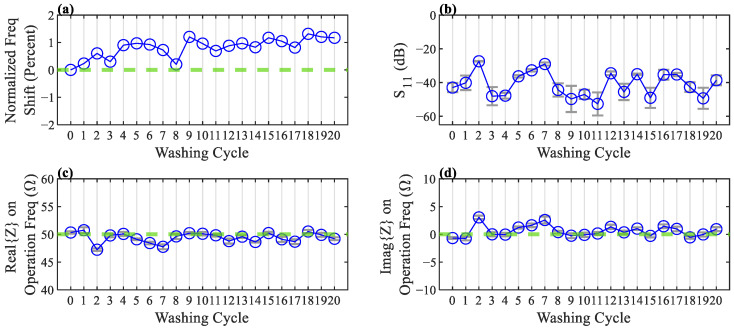
Antenna electrical characteristics are measured after each washing cycle. Each measurement is made four times, and the average is reported. Error bars show the standard deviation of each measurement. (**a**) Normalized frequency shift of the antenna. Green dashed line indicates zero shift. (**b**) Return Loss of the antenna on resonance frequency. (**c**,**d**) Real and Imaginary part of the antenna’s impedance on operation frequency, respectively. Green dashed line indicates the ideal values for a 50 Ω impedance matching.

The measurements after each cycle are shown in Figure 12. It can be seen that there is a slight shift in the antenna’s operation frequency throughout the cycles, which could be due to the antenna deformations or fabric shrinkage caused by the heat during the drying process. The reflection loss of the antenna is still ideally below −10 dB, and despite the slight frequency shift, the antenna’s performance is not significantly affected after 20 wash cycles.

## 6. Discussion

### 6.1. Strain Sensitivity

The introduced antenna design is notably valuable in strain sensing applications. It was previously shown in Figure 3 that the impedance and the resonance frequency of the antenna shift as SR changes when the sinusoidal dipole becomes stretched or compressed along its axis. One can exploit this behavior and make a sinusoidal antenna out of flexible conductors and record the S-parameters of the antenna over time as an indicator of the strain applied to the antenna [4]. The strain detection can be achieved by tracking the resonance frequency or measuring the reflection coefficient on a fixed frequency.

**Figure 13:**
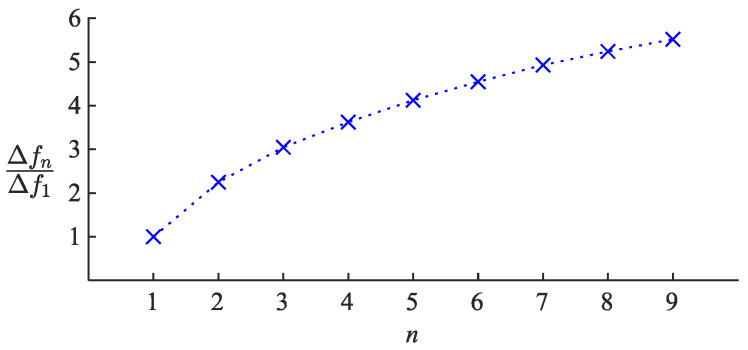
Sensitivity of the resonance frequency of the sinusoidal antenna sensor to the applied strain, expressed relative to the sensitivity of the traditional antenna sensor with n=1, for antennas n>1.

The traditional antenna sensor employed in previous works on respiration monitoring [2,3,4] is essentially a sinusoidal half-wave dipole n=1. Higher n values can increase the sensitivity of the antenna sensor. For comparison, by choosing n=2, the resonance frequency sensitivity of the antenna versus SR will be ≈2.2 times more than the sensitivity demonstrated by the traditional antenna. It increases to ≈4.1 and ≈5.5 times more for the antennas n=5 and n=9, respectively, which is a significant improvement. Figure 13 shows the trend of sensitivity improvement for different n values.

Although a higher n value can provide better sensitivity, the advantage is reduced as n become larger and larger. For instance, raising n from 7 to 9 only improves the sensitivity by 12%, while changing n from 2 to 4 will boost the sensitivity by a significant amount of 83%. Moreover, an antenna sensor designed with a very high n value could be challenging to fabricate if the wire is relatively thick, and it will not be easy to integrate the antenna into wearable applications.

Table 4 summarizes the characteristics of this work compared to other recently published works on flexible antennas for strain sensing. Stretching strain is defined as $\epsilon =\Delta L/L_{0}$ where $L_{0}$ is the initial antenna sensor length, and stretchability is $\epsilon_{max}\times 100\%$ which is the maximum amount of the possible strain ϵ that can be applied to the sensor, expressed in percentage [7]. Bending strain is defined as ϵ=h/2r where h is the antenna sensor thickness and r is the bending radius. The maximum bending is defined as the angle at which the sensor cannot be bent any further [11]. Exceeding the limits of the reported stretchability (or maximum bending) is either destructive for the antenna sensor or yields unreliable sensor readings [7,11]. The sensitivity to strain is also defined as $S=\left(\Delta f/f_{0}\right)/\epsilon$ which is the normalized frequency shift $\Delta f/f_{0}$ for an applied strain of ϵ [7,11]. Table 5 compares recent works on another category of antenna strain sensors primarily used in structural health monitoring to detect surface strains and cracks in the structures.

A crucial factor for antenna sensor comparison is the magnitude of force needed to achieve a specific strain. This factor becomes especially important in applications with very small stretching forces. To the best of our knowledge, such a parameter is not reported in any of the works summarized in Table 4. However, the materials used in the fabrication of each antenna sensor are well described in the other works and this paper, which might indirectly address this issue. Nonetheless, a study on the magnitude of force required to achieve a specific strain on the antenna introduced here is undoubtedly an interesting point that could be experimentally measured and included in a forthcoming paper.

### 6.2. Effects of Bending and Twisting

**Figure 14:**
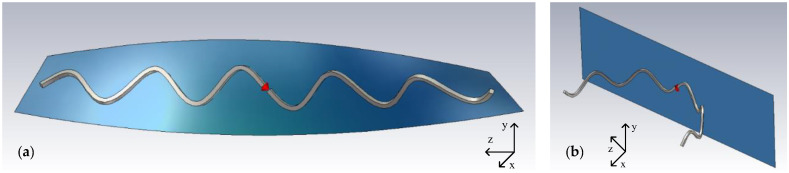
Illustration of (**a**) the twisting and (**b**) the bending applied to the modeled antenna in simulation software. The blue metallic-colored plane, the thick wire, and the lighting effects are added for a better presentation of the 3D model.

Although the antenna sensor introduced here is ideally designed for applications where the deformations are mainly along the *Z*-axis, the effects of other forms of deformations are also investigated to understand the antenna’s behavior better. A sample 50 Ω sinusoidal antenna with the geometrical parameters introduced in Table 2 is simulated in twisted and bent conditions, as illustrated in Figure 14. The twisting is performed around the *Z*-axis up to $90^{{}^{\circ}}$, and the cylindrical bending is applied along the *X*-axis up to $180^{{}^{\circ}}$.

**Figure 15:**
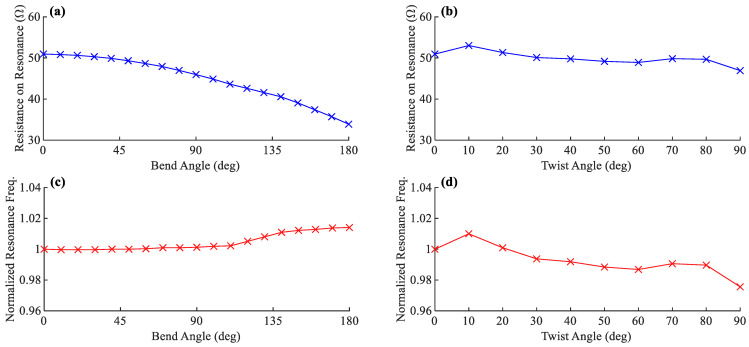
(**a**,**b**) The shift in antenna’s resistance on resonance and (**c**,**d**) its normalized resonance frequency for different angles of bending and twisting, respectively.

Figure 15 presents the antenna simulation results for different bend and twist angles. There is a significant change in the antenna’s resistance on the resonance frequency under bending deformation. It is shown that the more the antenna becomes bent, the lower becomes the resistance on the resonance frequency. Bending can also shift the resonance frequency to slightly higher, which is negligible in low bending angles and will be less than 2% for $180^{{}^{\circ}}$. In comparison, twisting does not significantly affect the resistance on resonance and shows a slightly decreasing trend. It also has a similar effect on the resonance frequency, making it drop by as much as 2.5% at $90^{{}^{\circ}}$ of twisting according to the simulation.

### 6.3. Specific Absorption Rate (SAR) Analysis

The amount of radio frequency (RF) radiation exposure is a critical factor that should be controlled in the radiating systems embedded in home appliances and portable devices. This concern becomes even more important in wearable devices and smart textiles due to the continuity of radiation and the close proximity of the radiating elements to body tissues. The amount of RF exposure is regulated using a metric named specific absorption rate (SAR), which is the time derivative of an incremental amount of energy dissipated in a specific mass of tissues [44]. The Federal Communications Commission (FCC) defines the limit of the SAR level of mobile phones for public exposure to 1.6 W/Kg, averaged over 1 g of tissue [45].

**Figure 16:**
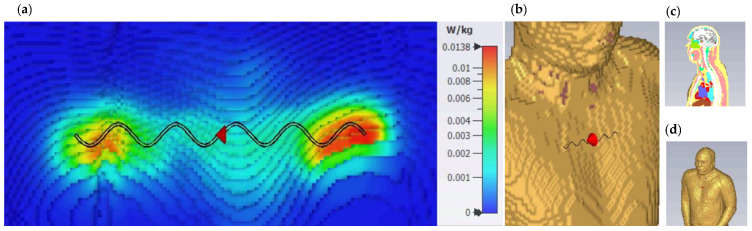
(**a**) SAR for excitation of 1 mW averaged over 1 g of tissue (**b**) Antenna placed on the chest area (**c**) Cross-section of the phantom model (**d**) Perspective view of the phantom model.

An analysis of SAR level is made on a sinusoidal antenna with n=5 and designed for 50 Ω on its 800 MHz resonance frequency. The antenna model is simulated while placed over the chest of the human body phantom model named Hugo from the CST Voxel Family, with a voxel resolution of 2 mm×2 mm×2 mm [31]. The gap between the antenna and the phantom model is considered 2 mm. The calculated SAR level for a reference excitation power of $P_{ref}=1\;\mathrm{mW}$ (equivalent to 0 dBm), averaged over 1 g of tissue, has a maximum value of 0.0138 W/Kg. Therefore, the maximum possible excitation power $P_{max}$ complying with FCC SAR level limitations can be calculated using a simple ratio as shown in Equation (12). Based on this calculation, the excitation power must be less than $P_{max}=115.9\;\mathrm{mW}$ (equivalent to 20.64 dBm) to comply with the limitations. Figure 16 presents the body phantom model and the SAR simulation results.

(12) $$\begin{array}{c} P_{max}=P_{ref}\cdot \left(\frac{SAR_{limit}}{SAR_{calculated}}\right)=1\;\mathrm{mW}\cdot \left(\frac{1.6\;W/\mathrm{Kg}}{0.0138\;W/\mathrm{Kg}}\right)\;\approx 115.9\;\mathrm{mW}\;\equiv 20.64\;\mathrm{dBm} \end{array}$$

## 7. Conclusions

A new flexible dipole antenna with a sinusoidal geometry is introduced. The radiative and electrical specifications of the antenna and the effect of geometrical parameters are presented. The antenna is fabricated using a biocompatible conductive polymer with high flexibility and great endurance. In contrast to the previous generation of antenna sensors made from glass-based material, the new antenna will not break by twisting, bending, and stretching, and can be used in wearable applications without compromising the user’s comfort. It is also shown that the new antenna design can have up to 5.5 times more sensitivity than the traditional antenna sensor employed in the previous works for respiration monitoring [2].

Additionally, a design guide for sinusoidal antennas is provided, and curve fittings are performed to estimate the geometrical factors based on the radiation characteristics of choice. The equations presented here provide a starting point for fine-tuning the geometrical parameters to achieve the desired radiation characteristics. Finally, a sample antenna is designed and fabricated in two versions, one with copper and one with the conductive polymer. The fabrication method is described, and the electrical specifications of the fabricated antennas are reported in free space and over the body. It is shown that there is a good agreement between the simulation and the measurements. Experiments are performed to prove the durability of the antenna sensor for everyday use, and it is shown that the performance of the antenna does not decay after 20 washing, rinsing, and drying cycles.

The contributions of this paper were mainly in the antenna design, characteristics, and strain sensitivity analysis. It was shown that the new antenna could provide better performance in sensing and be a superior choice for wearable applications compared to its previous generation due to its miniature size, flexibility, and durability. An upcoming paper will be focused on the application of the introduced antenna in a vital signal monitoring system. The antenna’s performance in sensing applications will be evaluated, and the measurement data will be provided.

## Figures and Tables

**Table 1:** The Maximum Gain and HPBW of the straight half-wave dipole and 50 Ω sinusoidal dipoles for different n values.

n	Max Gain (dBi/dBd)	HPBW XZ Plane (ϕ=0°)	HPBW YZ Plane (ϕ=90°)
0 *	2.14/0	78°	78°
1	2.419/0.269	89.6°	82.6°
3	2.451/0.301	79.2°	80.6°
5	2.467/0.317	78.5°	79.7°
7	2.474/0.324	78.3°	80.1°

* Indicates traditional straight half-wave dipole.

**Table 2:** Geometrical Parameters of the antennas.

Parameter	$\mathrm{Axial}\;\mathrm{Length}\;(L_{A})$	$\mathrm{Width}\;(W_{A})$	n	Wire Thickness
Value	74.54 mm	5.65 mm	5	1.4 mm

**Table 3:** Measured Antenna Specifications.

Antenna	Operation Frequency	Impedance on Frequency
Copper	790 MHz	52+0.2 i Ω
Conductive Polymer (Free space)	827 MHz	50.4+2.5 i Ω
Conductive Polymer (Over the Body)	808 MHz	49.2+19.9 i Ω

**Table 4:** Comparison of recent works on stretchable antennas for strain sensing.

Description	Strain Type	Stretchability/Max. Bending	Sensitivity to Strain	Year	Ref.
Sinusoidal dipole antenna *	Stretching	30%	0.40	2022	This work
Serpentine meshed patch over ground plane	Stretching	40%	0.20	2021	[7]
Serpentine meshed in patch and ground planes	Stretching	100%	0.25	2021	[7]
RFID Meandered half-wave dipole in Ecoflex	Stretching	Not provided	0.141 **	2019	Based on [8]
Flexible planar dipole antenna over Kepton tape	Stretching	Not provided	0.066 **	2012	Based on [42]
Liquid metal loop antenna	Stretching	40%	0.18	2009	[43]
Graphene patch antenna over copper tape	Bending	Not provided	1.4	2021	[9]
Flexible multi-layer graphene film	Bending	Not provided	5.39	2018	[10]
Aluminum tape patch over cellulose substrate	Bending	$160^{{}^{\circ}}$	3.49	2016	[11]

* With the assumption of n=9 and the initial SR=0.3. In the case of using adequately thin wires, a higher initial SR and therefore higher stretchability could be achieved. ** The original author does not provide the sensitivity directly. The presented value is calculated from the reported data.

**Table 5:** Comparison of recent works on antenna sensors for structural health monitoring.

Description	Resonance Freq	Sensitivity to Strain (kHz/με) *	Year	Ref.
Double split-ring resonator (dSRR) antenna	2.725 GHz	−1.548	2022	[16]
Rectangular microstrip antenna	2.725 GHz	−2.379	2022	[16]
Rectangular microstrip antenna	2.469 GHz	−2.847	2021	[12]
Sierpinski fractal microstrip patch	2.725 GHz	−1.18	2019	[15]
Circular patch antenna	2.5 GHz	−2.05	2018	[13]
RFID folded patch antenna	911.6 MHz	−0.76	2015	[14]

* The sensitivity of this category of antenna sensors is usually reported in absolute frequency.

## Appendix A

The correlation between the simulated data and the introduced fitted equations is illustrated in this appendix for both design methods based on SR and WR.
